# Same family, same mutation, different ECG

**DOI:** 10.1002/mgg3.2079

**Published:** 2022-10-28

**Authors:** Kürşat Akbuğa, Mustafa Karanfil

**Affiliations:** ^1^ Cardiology Department, Faculty of Medicine TOBB ETU Ankara Turkey; ^2^ Cardiology Clinic Ankara City Hospital Ankara Turkey

**Keywords:** electrocardiography, long QT syndrome, mutation

## Abstract

**Background:**

Different types of long QT syndromes (LQTS) have distinct ECG manifestations according to the type and magnitude of ion channel dysfunction. While LQT1 carriers usually have broad‐based T waves and LQT3 carriers have extended ST segments with relatively narrow peaked T waves; LQT2 carriers have low‐amplitude T waves with high incidences of notches.

**Methods:**

We describe three members of a family with the same LQTS2 pathogenic variant, but different surface ECG findings.

**Conclusion:**

This case shows ECG differences may also occur between family members who have pathogenic variants associated with long QT syndrome.

## Graphical abstract

This case shows ECG differences may also occur between family members who have the same gene mutation due to long QT syndrome.

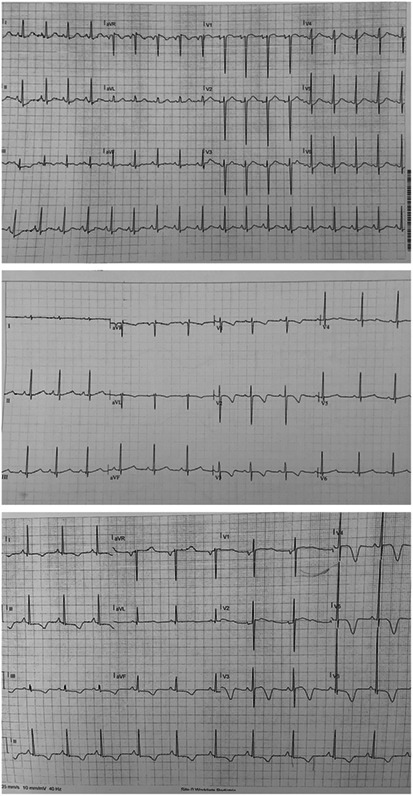

## 1 INTRODUCTION

The congenital long QT syndrome (LQTS) is a life‐threatening cardiac arrhythmia syndrome that represents a leading cause of sudden death in the young. LQTS is typically characterized by a prolongation of the QT interval on the ECG and by the occurrence of syncope or cardiac arrest, mainly precipitated by emotional or physical stress (Schwartz et al., 2012).

By far, *KCNQ1* (LQT1), *KCNH2* (LQT2), and *SCN5A* (LQT3) are the most common LQTS genes, accounting for ≈90% of all genotype‐positive cases (Kapplinger et al., 2009).

As is known, QT duration is not used to differentiate long QT syndromes. Long QT syndromes with the highest prevalence (LQTS1, LQTS2, and LQTS3) were found to have their own ECG characteristics. LQT1 generally has a broad‐based T wave. LQT2 is generally characterized by low‐amplitude T waves, while LQT3 is characterized by peaked T waves (Zareba, 2006).

## 2 CASE PRESENTATION

A 44‐year‐old male patient diagnosed with LQTS2 was admitted to the cardiology outpatient clinic for routine clinical control. He had two syncopal episodes more than a decade ago. As he remembered, both episodes were while he was suffering from a cold and on medications. After diagnosis, he was followed up without any cardiac device but with beta‐blocker(propranolol) and with the advice of not to use QT‐prolonging drugs. He had no syncopal episodes for 10 years now. His complete blood count and biochemistry results were in the normal range. His echocardiography revealed normal systolic functions with normal wall thickness. Also, there was no valve dysfunction.

When his medical history was deepened and genetic reports were examined, the following were found: First, *SCN5A, KCNQ1*, and *KCNH2* genes were examined in the whole gene sequence analysis (exons, exon–intron junctions, and promoter region) performed on the father. After the detection of the KCHN2 mutation, sequence analysis (known mutation, 4th exon) analysis of the *KCNH2* gene was performed on both children and the same variant was detected.

**FIGURE 1:**
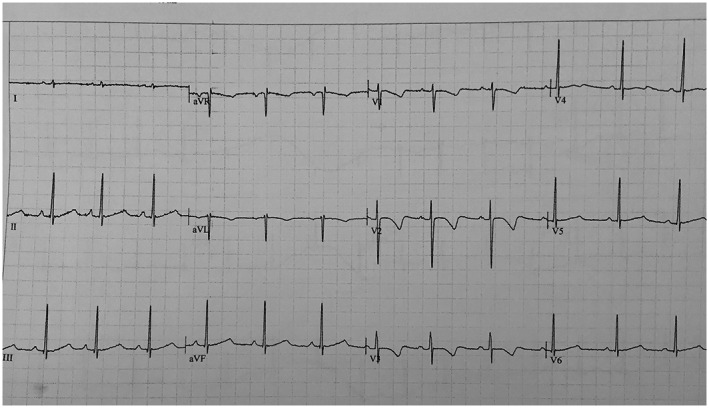
ECG of Daughter

**FIGURE 2:**
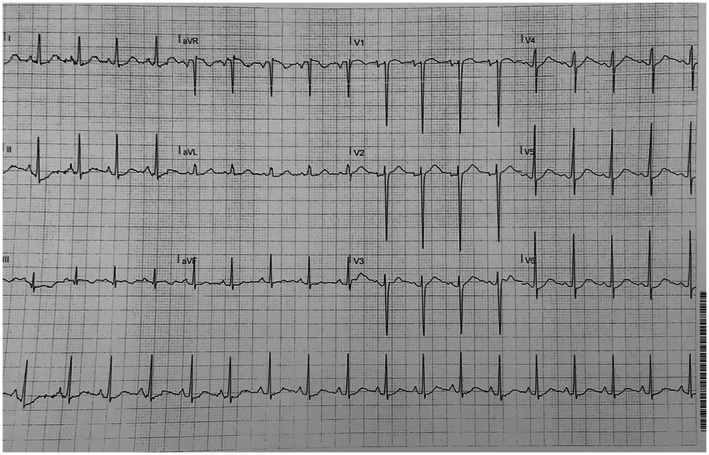
ECG of Son

**FIGURE 3:**
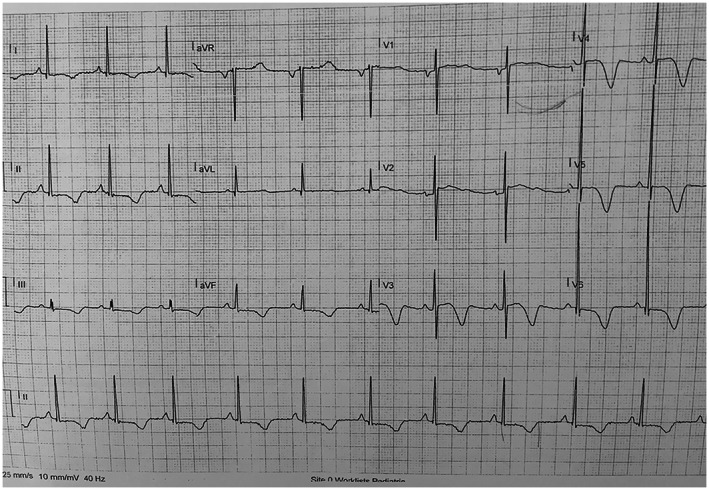
ECG of Father

The patient's daughter had QTc of 580 ms (Figure 1) and his son also had QTc of 480 ms (Figure 2). Our patient's QTc was 510 ms during rest (Figure 3).

While her daughter and son had low amplitude and notched T waves, our patient had deep negative T waves.

According to the molecular genetic analysis, all three members of the family had exactly the same mutations (NM_000238.3, p.S207Afs*9, and c619_619delA) in the *KCHN2* gene. According to the genetic report, although the mutation detected in the *KCNH2* gene is a previously unidentified mutation, it was considered as the cause of the disease due to the frameshift and early stop codon according to the “mutation taster” evaluations. Also, the genetic report stated that it was recommended to investigate the same mutation in the patient's siblings, parents, and children, if any. There was an indication for preimplantation genetic diagnosis in subsequent pregnancies of the family.

We decided to continue his medication and follow‐up as before.

## 3 DISCUSSION

Data indicate that according to T‐wave morphology, we can often distinguish between hereditary L‐QT syndromes.

In the study of Strujnik and his colleagues, ECGs were analyzed according to the T‐wave morphology in terms of duration, asymmetry, flatness, and amplitude. The study revealed that similar T‐wave morphology was present in individuals with the same LQTS types (Struijk et al., 2006). Also in the study of Zhang and his colleagues, it is concluded that most genotyped patients with LQT2 had characteristic ST‐T‐wave patterns that could be used to identify mutation‐positive patients (Zhang et al., 2000). In a study of 230 LQT patients (110 of them LQT2), the overall predictive accuracy of ECG‐guided genotyping was 78% for LQT2 (Gao et al., 2016).

Contrary to these findings, our patient and his children had different ECG patterns despite the same mutations.

## 4 CONCLUSION

Although age, gender, and beta‐blocker use can affect ECG manifestations of the same mutations; to the best of our knowledge, we have not previously seen this degree of variability in ECG patterns within the same family with the same LQT2 variant.

ECG differences may also occur between family members who have the same genetic variant causing long QT syndrome. Surface ECG is not sufficient to definitively type patients with long QT syndromes. Genetic analysis is important for guiding the treatment.

## AUTHOR CONTRIBUTIONS

Mustafa Karanfil collected the clinical data. Kürşat Akbuğa drafted the initial manuscript. Both authors interpreted results and reviewed the manuscript. Kürşat Akbuğa revised the manuscript. All authors agreed to accept responsibility for this work and agreed to the final manuscript as submitted.

## FUNDING INFORMATION

None.

## CONFLICT OF INTEREST

On behalf of all authors, there is no conflict of interest.

## ETHIC STATEMENT

The written informed consents were obtained from all 3 patients. As it was a case report, it was not presented to the ethics committee.

## Data Availability

Data available on request from the authors.
